# Analyses of methylation status of CpG islands in promoters of miR-9 genes family in human gastric adenocarcinoma

**Published:** 2015-06

**Authors:** Raziyeh Ebrahimi-Askari, Mehrdad Behmanesh, Masoud Soleimani

**Affiliations:** 1 Department of Genetics, Faculty of Biological Sciences, Tarbiat ModaresUniversity, Tehran, Iran; 2 Department of Hematology, Faculty of Medicine, Tarbiat Modares University, Tehran, Iran

**Keywords:** Gastric cancer, Epigenetic, DNA methylation, miR-9, MS-PCR

## Abstract

In the recent years deregulation for microRNAs expression pattern have emerged as a possible molecular factor for carcinogenesis. It has been reported that the expression of miR-9 was down-regulated in human gastric adenocarcinoma. To figure out the molecular mechanism of this down regulation, the methylation status in promoters of miR-9 family loci were compared in the human gastric adenocarcinoma samples with their normal margins. Using a methylation specific PCR technique the methylation status of miR-9 family loci were compared between 30 pairs of primary human gastric adenocarcinoma samples with their normal margins. The methylation of miR 9-1 status showed no specific difference in promoter methylation pattern in tumor and normal specimens, while in the miR-9-2 locus were unmethylated in both types of tissues. The promoter of miR-9-3 locus seems to be specifically methylated in tumor and their normal margin tissues. Our data revealed methylation of these CpG islands were not meaningfully different between normal and tumor gastric adenocarcinoma specimens and the methylation status of promoter may not be able to account for alteration of miR-9 expression in this type of gastric cancer.

## INTRODUCTION

Gastric cancer is the forth-commonest malignancy in the world. Over 95% of malignancies of the stomach are adenocarcinomas [1]. Previous studies have revealed that deregulation in some microRNAs gene expression is related to gastric cancer development or progression [2-4]. MicroRNAs (miRNAs) are evolutionary conserved, small single-stranded noncoding RNAs between 18-25 nt in length that synthesized in the nucleus by a RNA polymerase II, via several intermediates by cleavage of much longer hairpin-shaped precursor (70-100 nt) transcripts. miRNAs are among small noncoding RNAs that regulate target gene expression through an almost perfect pairing mainly with 3’ untranslated regions (UTRs) of target mRNAs, inducing direct mRNA degradation or mediate post-transcriptional gene silencing [5-6].

Recently it is shown that miRNAs have critical role in development, proliferation and apoptosis, as well as, involvement in the pathogenesis of many human diseases and cancers. Interestingly, it is shown that in human cancers miRNAs expressions differ between normal and tumor tissues, as well as, different tumor types [7-8]. It has been suggested that down-regulation of some miRNAs gene expression in tumors in comparison with their normal tissues demonstrated their putative tumor suppressor roles [7-10]. Epigenetic mechanisms including DNA methylation and histone modifications are involved in miRNAs genes expression and regulation [11-14].

miR-9 is one of the conserved microRNAs among different organisms found in insects to mammals. In human, Hsa- miR-9 is transcribed from three genomic loci including 1q22 (miR-9-1), 5q14.3 (miR-9-2) and 15q26.1 (miR-9-3). In transcription, these three loci produce different hairpin precursors but they have same mature sequences in the final processed form. The promoters of these loci are embedded in CpG islands. It has been shown that this miRNA has a tissue-specific expression pattern in neuronal tissues, in normal condition to regulate their normal development [15-19]. Deregulation of miR-9 genes expression are involved in pathogenesis of some important human diseases such as cancer in colorectal, gastric, breast, non-small cell lung and even cardiac hypertrophy [20-23]. In some of cancers the promoter hyper-methylation of miR-9 had correlation with metastasis formation [15, 24].

There are some controversial studies about different result for the expression pattern of this miRNA and their promoter status methylation in different cancers. miR-9 is overexpressed in several cancer forms, such as brain tumors, hepatocellular carcinomas and Hodgkin lymphoma (HL). The up-regulation of miR-9 expression level in breast cancer cells lead to increase cell motility, invasiveness and tumor angiogenesis through activation of β-catenin signaling pathway. The overexpression of miR-9 in non- metastatic breast tumor cells causes these cells to make pulmonary micrometastasis in mice and its inhibition in highly malignant cells prohibit metastasis formation. In early breast cancer and colorectal cancer miR-9 was transcriptionally downregulated in a methylation dependent way [17-18].

It has been reported that miR-9 is downregulated in gastric carcinoma and it has tumor suppressor activity by NF-κB1 expression regulation [10, 19], we reasoned that hypermethylation of CpG islands located in the promoter of one or more miR-9 genomic loci in cancerous tissue might be responsible. In the present study the methylation status of CpG islands located in miR-9-1, miR-9-2 and miR-9-3 genomic loci in thirty derived adenocarcinoma tumors and their normal margin tissues as well as human gastric adenocarcinoma cell line AGS, are compared.

## MATERIALS AND METHODS

Thirty gastric cancer specimens and their paired normal marginal tissues were obtained from Iran Tumor Bank (Tehran, Iran). The samples consisted of 19 males and 11 females with 62±9.8 years. The tissues were immediately snap-frozen in liquid nitrogen and kept in -86°C till molecular analysis. Small parts of sample were used for histopathological analysis by two independent pathologists. The evaluation was according to the World Health Organization parameters for grading and TNM system for stage classification. Written informed consent was obtained from the participants prior to sampling. The Ethics Committee of Tarbiat Modares University approved the experiment design.

Human gastric adenocarcinoma cell line AGS was purchased from the National Cell Bank of Iran (Pasteur institute, Tehran, Iran). AGS cells were cultured in Dulbecco’s modified Eagle’s medium (DMEM, Gibco) supplemented with 10% (V/V) fetal bovine serum (Gibco), 100 U/ml penicillin and 100 µg/ ml streptomycin, maintained at 37°C in a humidified atmosphere with 5% CO2.

Total genomic DNA was isolated from fresh frozen biopsies, blood samples andfrom cultured cells by TRIZOL reagent (Invitrogen, Carlsbad, Ca, USA) according to the manufacturer’s instruction. The quality and concentration of the extracted genomic DNAs were examined spectrophotometrically and visualization after electrophoresis in1% agarose gel.

To do Bisulfite treatment of genomic DNA two microgram of extracted DNA from a cancerous, their normal margin tissues and cultured cell were subjected to biosulfite conversion using EpitTect Bisulfite Kit (QIAGEN). In this modification methylated DNA is protected and unmethylated cytosine is converted to the uracil. Briefly, the DNA in bisulfite conversion mixture was pre-incubated at 60°C for 10 min in a total volume of 140 µl and the conversion reaction was done by thermal cycler conditions according to manufacturer's instruction. The purified DNAs were stored at -20°C till molecular analyses.

For analysis of CpG island methylation status of mir-9 family, EpiTect Control DNA (human), universal methylated DNA (Chamicon International, Inc), and normal lymphocytes DNA were used as controls for discriminating of methylated and unmethylated alleles, respectively. Converted genomic DNA of tumors and their normal margins served as a template using specific primers set for the methylated or modified methylated sequences [15]. MSP amplification was performed in 25 µl reaction mixture containing 10mM Tris-HCl, 50 mM KCl, 1.25 mM MgCl2, 2 µl of bisulfite modified DNA, 0.5 U of recombinant Taq DNA polymerase (Cinnagen), 4 µM of each primer, and 200 µM of each deoxynucleoside triphosphate. The initial denaturation was performed at 95°C for 3 min and amplification was performed for 40 cycles of denaturation at 95°C for 30 sec, annealing 55 or 57°C (dependent on used primers) for25 sec, and extension at 72°C for 60 sec, followed by a final extension at 72°C for 10 min. The resultant PCR products were separated on 12% polyacryl amide or 2% agarose gel stained with ethidium bromide and visualized under UV illumination.

To determining of correlations between methylation status and clinopathological features *x*^2^ or Fisher’s exact probability tests were used. All P values presented are two- sided. A P≤0.05 was regarded as statistically significant. Statistical analysis of the data was performed using SPSS software version 18 (Chicago, IL, USA).

## RESULTS

**Figure 1:**
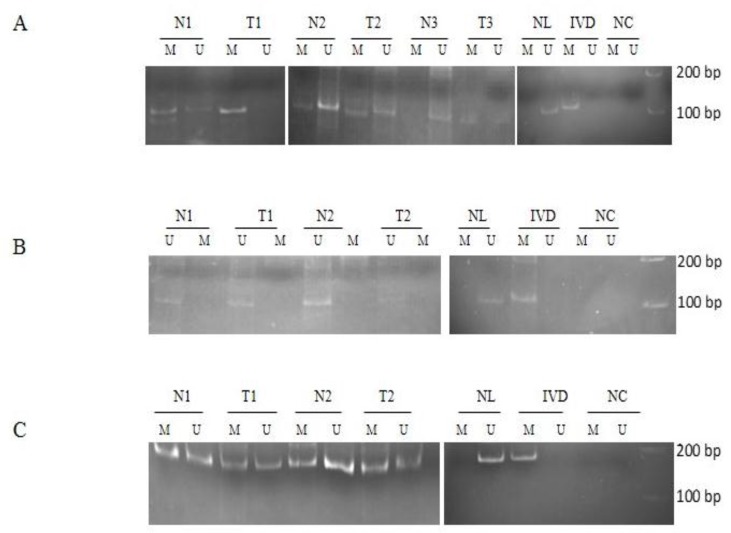
Methylation-specific PCR analyses of promoters in miR-9 loci family in tumors (T) and their normal (N) tissues. Part (A) represent the MSP analyses of miR-9-1, part (B) MSP analyses of miR-9-2 and part (C) MSP analyses of miR-9-3 loci, respectively. The presence of a band under the U or M lanes indicates unmethylated or methylated sequences. Genomic DNA from normal lymphocytes (NL) and *in vitro* methylated DNA (IVD) are shown as positive controls for the unmethylated and methylated sequences, (N) Normal gastric tissue, (T) Tumoral gastric tissue, (NC) Negative Control, ( L) DNA ladder

The methylation status of the CpG islands of miR-9 loci by MSP in samples of gastric cancer patients were analyzed in comparison to their normal margins. The miR-9-2 promoter was unmethylated in both normal samples and tumors (Fig. 1B); but miR-9-3 showed methylation of one allele and unmethylation of the other one in all of specimens (Fig. 1C). However, miR-9-1showed methylation of at least one allele in73.3% of normal samples and 76.6% in tumor samples, respectively (Fig. 1A). Methylation status of miR-9-1 CpG island was not meaningfully different between tumor and normal samples (P=0.85). Summary of miR-9- 1, miR-9-2 and miR-9-3 promoter analyses result by MSP in primary gastric adenocarcinoma and normal samples are presented in Table 1.

With regard to the variation in methylation status of mir9-1 with mir9-2 and 3 promoters in tumors and their normal margins, we investigate for the possible correlation with the clinopathological features of specimens. There was no significant correlation between miR-9-1 methylation status and clinopathological features of tumor samples (Table 2).

**Table 1:** Summary of miR-9-1, miR-9-2 and miR-9-3 promoter methylation analysis results

	**miR9-1**		**miR9-2**		**miR9-3**	
	**Non-tumor**	**Tumor**	**Non-tumor**	**Tumor**	**Non-tumor**	**Tumor**
**M/M**	12	14	0	0	0	0
**U/U**	8	7	30	30	0	0
**M/U**	10	9	0	0	30	30

Note: The methylation status of promoters was examined by methylation specific PCR technique. M and U are indicating methylated and unmethylated status of loci in promoter regions of mir-9 family, respectively.

**Table 2:** Analyzing of possible relation between clinopathological characteristics of patients with MiR-9-1 locus methylation status

** Characteristics**	**miR-9-1** ** methylated**	**miR-9-1** **unmethylated**	**P-value**
**Age**			0.66
>62	9	4	
<62	14	3	
**Gender**			1.00
Male	15	4	
Female	8	3	
**Tumor stage**			0.64
I+II	6	3	
III+IV	17	4	
**Tumor grade**			0.20
I+II	9	5	
III+IV	14	2	
**Lymph node metastasis**			1.00
No	11	1	
Yes	12	6	
**Distant metastasis**			0.19
No	15	5	
Yes	8	2	

**Figure 2:**
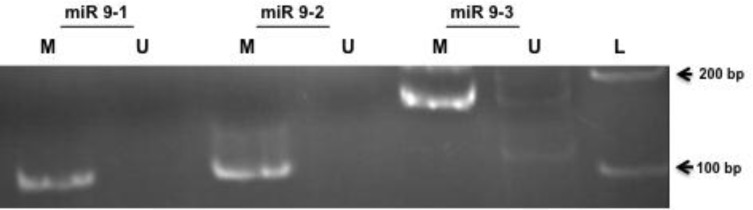
Methylation-specific PCR analyses of miR-9 in AGS cell line. The presence of a band under the U or M lanes indicates unmethylated or methylated sequences, respectively and L is presenting DNA ladder.

In AGS cell line miR-9-1 and miR9-2 loci were fully methylated. However miR-9-3 locus was methylated in one allele and unmethylated in the other one in AGS cell line (Fig. 2). Our data revealed that methylation of these CpG islands were not meaningfully different between normal and tumor gastric adenocarcinoma specimens.

## DISCUSSION

Recent reports revealed that some miRNAs were deregulated in human malignancies [21-24]. Saito et al first addressed epigenetic regulation of miRNAs. They identified those 17 miRNAs up regulated in bladder cancer after treatment with 5-aza and PBA [11]. Identification of cancer specific miRNAs and molecular mechanisms underlying regulation of miRNAs expression may be important for defining novel targets for cancer therapies [25-26].

MiR-9 expression level increases during embryonic stem (ES) cell differentiation to neural precursors [27]. MiR-9 acts as a putative tumor suppressor gene on recurrent ovarian cancer and inhibits ovarian cancer cells growth through NF-κB1 regulation [28,29]. Luo et al reported miR-9 down-regulation in gastric carcinoma [19]. Wan et al showed miR-9 is down-regulated in gastric adenocarcinoma and inhibits the growth of human gastric adenocarcinoma cell line MGC803 through NF-κB1 gene regulation [20]. To figure out the cause of reduction reduction in miR-9 expression of human gastric cancer, we examined the methylation status of the CpG islands of miR-9 loci in human gastric primary tumors with normal margin gastric tissue and AGS cell line.

Since miR9-2 CpG island was unmethylated in all of the normal and tumor patient samples we reasoned that transcription of this site would not be affected by methylation. While in the contrary, miR-9-2 CpG island was methylated in the AGS cell line. miR-9-2 methylation in AGS cell line reveals that all of the molecular alteration of cancerous cell line cannot be attributed to matched in situ tumors. Ertel et al. showed differential in methylation pattern using cellular pathways by tumor cells and cancer cell lines [30].

miR9-3 CpG island showed methylation in one allele and unmethylation status in the other one in all of patient samples and AGS cell line too. The observed heterozygous methylation status of miR9-3may be an allele-specific methylation (ASM) phenomenon.

In 2008, Kerkel et al. showed that tissue specific ASM outside of imprinted genes showing strong correlation with local SNP genotypes [31]. Two recent genome wide surveys of ASM showed statistically significant CpG SNPs near loci with ASM [32,33]. Since ASM usually is due to cis-effects of existed genetic polymorphisms it is possible that a CpG SNP variation may be near to the miR-9-3 CpG island locus. However, the methylation status of miR-9-1 CpG island was not meaningfully different between tumor and marginal samples. There was no correlation between miR-9-1 methylation and clinopathological features of tumors.

Based on two studies miR-9 is down-regulated in gastric carcinoma and has tumor suppressor activity by NF-κB1 expression regulation [18-19], but recently it has been reported that miR-9 is up-regulated in gastric cancer tissues and down-regulated CDX2 expression but methylation status of these three miR-9 regions did not correspond to the expression levels of precursor miR-9 or maturemiR-9 [34]. Therefore the exact function of miR-9 as a tumor suppressor, oncogene or both of them, remains to be elucidated in gastric cancer. Aberrant hypermethylation of miR-9 family genes (miR-9-1, miR-9-2 and miR-9-3) was reported in some primary tumors with lymph node metastasis such as colon, lung and breast cancers, and melanoma [15].In early breast cancer and colorectal cancer miR-9 was transcriptionally down-regulated in a methylation dependent way [16,18].On the contrary, recently it is reported that miR-9 levels were significantly up regulated in primary breast tumors from patients with metastases compared to those from metastasis-absent patients. In the other hand Tsai et al reported that DNA methylation tightly repressed miR-9 through the simultaneous methylation of the CpG- rich regions of these three independent genes [35]. Butour data revealed that methylation status of miR-9 family CpG islands are not different between tumor and non-tumor tissues of gastric, and cannot account for alteration of miR-9 expression in this type of gastric cancer [34]. However one of the alleles of miR9-3 may be specifically methylated in gastric tissue. The difference between our data and Tsai et al data may be population based (population epigenetics).

Taken together, it seems that the exact function and regulatory mechanisms of miR-9 expressions remain to be elucidated in gastric cancer and further studies on miR-9 expression and methylation of their promoters in normal gastric and other cancer are needed.

## Notes

**Acknowledgements: **The authors gratefully acknowledge the contribution of the patients in this study. The Iranian Council of Stem Cell Technology and the Department of Research Affairs of Tarbiat Modares University provide the funding of this work.
